# Increased cell killing effect in neutron capture enhanced proton beam therapy

**DOI:** 10.1038/s41598-024-79045-3

**Published:** 2024-11-18

**Authors:** Shintaro Shiba, Takahiro Shimo, Masashi Yamanaka, Takayuki Yagihashi, Makoto Sakai, Tatsuya Ohno, Koichi Tokuuye, Motoko Omura

**Affiliations:** 1 Department of Radiation Oncology, Shonan Kamakura General Hospital, 1370-1, Okamoto, Kamakura, Kanagawa 247-8533 Japan; 2 Department of Radiation Oncology, Gunma University Graduate School of Medicine, 3-39-22, Showa-machi, Maebashi, Gunma Japan; 3 Radiological Research Division, Shonan Research Institute of Innovative Medicine, Shonan Kamakura General Hospital, 1370-1, Okamoto, Kamakura, Kanagawa 247-8533 Japan; 4 Department of Medical Physics, Shonan Kamakura General Hospital, 1370-1, Okamoto, Kamakura, Kanagawa 247-8533 Japan

**Keywords:** Proton beam therapy, Neutron capture enhanced proton beam therapy, Radiotherapy, Boron neutron capture reaction, Boron neutron capture therapy, Boronophenylalanine, Medical research, Oncology

## Abstract

Thermal neutrons generated in the body during proton beam therapy (PBT) can be used to cause boron neutron capture reactions and have recently been proposed as neutron capture enhanced PBT (NCEPBT). However, the cell killing effect of NCEPBT remains underexplored. Here, we show an increase in the cell killing effect of NCEPBT. Using Monte Carlo simulations, we showed that neutrons generated by proton beam irradiation are uniformly spread on tissue culture plates. Human salivary gland tumor cell line (HSG), human osteosarcoma cell line (MG63), human tongue squamous cell carcinoma cell line (SAS), and human malignant melanoma cell line (G-361) were irradiated with X-rays, proton beams, and proton beams with ^10^B-enriched boronophenylalanine (boron concentration of 20 and 80 ppm). The relative biological effectiveness (RBE) values of proton beams alone, proton beams with 20 ppm boron, and proton beams with 80 ppm boron for HSG, MG63, SAS, and G-361 were 1.02, 1.07, and 1.23; 1.01, 1.08, and 1.44; 1.05, 1.09, and 1.46; and 1.04, 1.13, and 1.63, respectively. NCEPBT with high boron concentration showed high RBE and a high sensitizing effect. Our results confirm an increase in the cell killing effect of NCEPBT, should aid in its clinical use, and warrant its further investigation.

## Introduction

Highly energetic proton beams, which are used in cancer treatment, have higher dose localization properties than X-rays used in radiotherapy (RT) owing to the distal tail-off due to the Bragg peak and sharp lateral^1–3^. This physical advantage enables high-dose administration of proton beams to the tumor while sparing normal tissue and may result in favorable outcomes for various cancer types^4–6^. Therefore, proton beam therapy (PBT) has become widespread in recent years. In contrast, PBT has a low linear energy transfer (LET) and is therefore less effective against radioresistant tumors than high LET radiotherapy such as carbon ion radiotherapy. Additionally, because PBT does not pinpoint irradiation at the cellular level, it can affect organs around the tumor and on the beam path, making it difficult to increase the administered dose beyond what is currently used in clinical practice.

Boron neutron capture reaction (BNCR) occurs in ^10^B, generating one alpha particle and one lithium ion: generating ^10^B (n, α) ^7^Li. Both particles have high LET and a range in biological tissues of approximately 5–9 μm, almost equal to the diameter of a tumor cell^7^. This reaction is exploited in boron neutron capture therapy (BNCT). Currently, boronophenylalanine (BPA) is used as the boron compound in clinical practice of BNCT, and BPA is selectively taken up by tumors through transporters that are specifically expressed in tumors, such as L-type amino acid transporter 1 (LAT1). Therefore, in BNCT with BPA, although BNCR occurs slightly in healthy tissues, it is more likely to occur in tumor cells expressing LAT1^8^.

In PBT, irradiation causes various nuclear reactions in the body and produces a mixture of fast and thermal neutrons^9,10^. This is quite distinct from the neutrons produced by collisions of the beam and beam-modifying devices. Thermal neutrons generated in the body during PBT can be used to trigger BNCR in boron-containing cells. Through BNCR, an increase in dose with high LET radiation at the cellular level can be expected, and it has the potential to improve the issues related with PBT in terms of therapeutic efficacy for radioresistant tumors and increased administration doses. Furthermore, neutrons generally are an unwanted secondary radiation that causes an extra dose absorption in patients and has potential long-term side effects^11^; however, BNCR via PBT could make use of the thermal neutron component to enhance the dose in tumors.

The concept of BNCR conducted by boron compounds and neutrons generated via PBT to improve the therapeutic effect of PBT has recently been proposed as neutron capture enhanced proton beam therapy (NCEPBT), and dose increases have already been confirmed and reported in Monte Carlo simulations^12^, however, the validation of NCEPBT biologically has not been reported. Here, we report a dose increase due to NCEPBT in our PBT system by Monte Carlo simulations and an increase in the cell killing effect of NCEPBT in several cancer cells confirmed in vitro.

## Methods

### Cell culture

In this study, we used a human salivary gland tumor (HSG) cell line, human osteosarcoma cell line (MG63), human tongue squamous cell carcinoma cell line (SAS), and human malignant melanoma cell line (G-361) obtained from Japanese Collection of Research Bioresources Cell Bank (JCRB). Cells were maintained in 10 cm tissue culture plates at 37 °C in a humidified atmosphere with 5% CO_2_, Dulbecco’s modified Eagle’s medium containing 10% heat-inactivated fetal bovine serum, and 1% penicillin-streptomycin. The medium and serum were purchased from Fujifilm Wako Pure Chemical Corporation (Tokyo, Japan). Cells were passaged before confluence and used for all experiments within 10 passages after purchase from JCRB to obtain stable results.

### Boron compounds

BPA, which was enriched with > 95% ^10^B, was provided by Stella Pharma Corporation (Osaka, Japan). BPA was combined in a medium with 10% molar excess of fructose (Fujifilm Wako Pure Chemical Corporation) to increase its solubility. Boron concentrations were rigorously measured using inductively coupled plasma optical emission spectroscopy (ICP-OES) (Spectrogreen, Hitachi-hightech, Tokyo, Japan) and adjusted to 20 and 80 ppm.

### Intracellular boron concentration measurement

Multiple dishes seeded with the same number of cells were prepared and incubated for at least 24 h. The cells were washed with phosphate-buffered saline (PBS) after removal of the medium, and exposure to BPA (boron concentrations of 20 and 80 ppm) for 2 h. Thereafter, BPA was removed, cells were washed with PBS, stripped from the dish using 0.25% trypsin-EDTA (Thermo Fisher Scientific, Waltham, MA, USA), and neutralized with the medium. Subsequently, cell counting was performed, and the medium was removed after centrifugation. The cells were then washed again with PBS and centrifuged again, and 2% sodium dodecyl sulfate (Fujifilm Wako Pure Chemical Corporation, Tokyo, Japan), as a surfactant, and 1 mol/L nitric acid (Junsei Chemical Corporation, Tokyo, Japan) were used to break the cell membrane and measure intracellular boron concentration using ICP-OES.

### Monte Carlo simulations

To confirm proton and thermal neutron fluence distribution, the Particle and Heavy Ion Transport code System (version 3.31) was used for Monte Carlo simulations^13^. Dose calculations were performed using the Japanese Evaluated Nuclear Data Library 4.0 developed by the Japan Atomic Energy Agency^14^, modeled on the nozzle section of our PBT system (PROBEAT-M1, Hitachi, Tokyo, Japan). The proton beam irradiation was performed using the spot-scanning methods with a spread-out of Bragg peak (SOBP) width of 6 cm, an energy range of 130.2–165.5 MeV, spot spacing of 5 mm, and field size of 20 × 20 cm. The 6-well tissue culture plates were positioned at 13.6 cm, which is the center of the SOBP, below the surface of a water-equivalent phantom (RW3, PTW, Freiburg, Germany) measuring 30 × 30 × 18.6 cm. The cell culture plate was placed in such a way that the center of the 1 mL medium coincided with the isocenter. This configuration reflected the conditions used in the measurements, with both the phantom and cell culture plate materials being replicated precisely. The thermal neutrons were defined as those with energies ≤0.6 eV^15^.

### X-ray and proton beam irradiation

X-ray irradiation was performed at the Shonan iPark (MBR-1520R-4, Hitachi, Japan). The dose rate and energy of X-ray irradiation were 1.7 Gy/min and 150 keV, respectively. Proton beam irradiation was performed at Shonan Kamakura General Hospital using the spot-scanning methods with an SOBP width of 6 cm, energy range of 130.2–165.5 MeV, a spot spacing of 5 mm, and field size of 20 × 20 cm. The physical dose of proton beam irradiation was calculated using the treatment planning system (VQA, Hitachi, Japan). The cells were irradiated with different doses (2, 4, 6, and 8 Gy) with both modalities. All experiments were performed in at least three replicates.

### Irradiation experimental procedures and clonogenic cell survival assay

Cells were seeded into 6-well tissue culture plates, 24–48 h before irradiation. Two hours before irradiation, the cells were exposed to BPA (boron concentration of 20 or 80 ppm) in a combination of proton beam irradiation and BPA groups, and only medium change was performed in the control group of proton beam or X-ray irradiation-alone groups; subsequently, the cells were exposed (or not) to X-ray or proton beam irradiation. After irradiation, the BPA-containing medium in the combination groups and BPA-free medium in the control groups were removed, cells were washed with PBS, and replaced with BPA-free medium.

After incubation for 10–14 d, the cells were fixed with methanol and stained with crystal violet. Colonies consisting of at least 50 cells were counted. Survival fractions were calculated as the ratio of surviving colonies per number of plated cells. Cell survival fractions were normalized to the survival fraction with the absence of irradiation (controls) in each group (proton beam irradiation alone, 20 ppm of boron with proton beam irradiation, 80 ppm of boron with proton beam irradiation, and X-ray irradiation alone). The dose that resulted in a surviving fraction of 10% (D_10_) was calculated using the linear-quadratic model. The relative biological effectiveness (RBE) values were obtained by comparing the D_10_ values for proton beam irradiation alone or proton beam irradiation, BPA, and X-rays. The sensitizer enhancement ratio (SER), as an indicator of the radiosensitizing effect of 20 or 80 ppm of boron, was calculated as the ratio of cell survival with proton beam irradiation alone to that with proton beam irradiation and BPA.

### Statistical analysis

The data from three independent experiments are expressed as the mean ± standard deviation. We determined the strength of associations between intracellular boron concentration and SER values using the Pearson correlation coefficient. Differences were statistically analyzed using a two-sided *t*-test. Statistical significance was set at *P* < 0.05.

## Results

### Monte Carlo simulations and neutron dose measurement

**Fig. 1:**
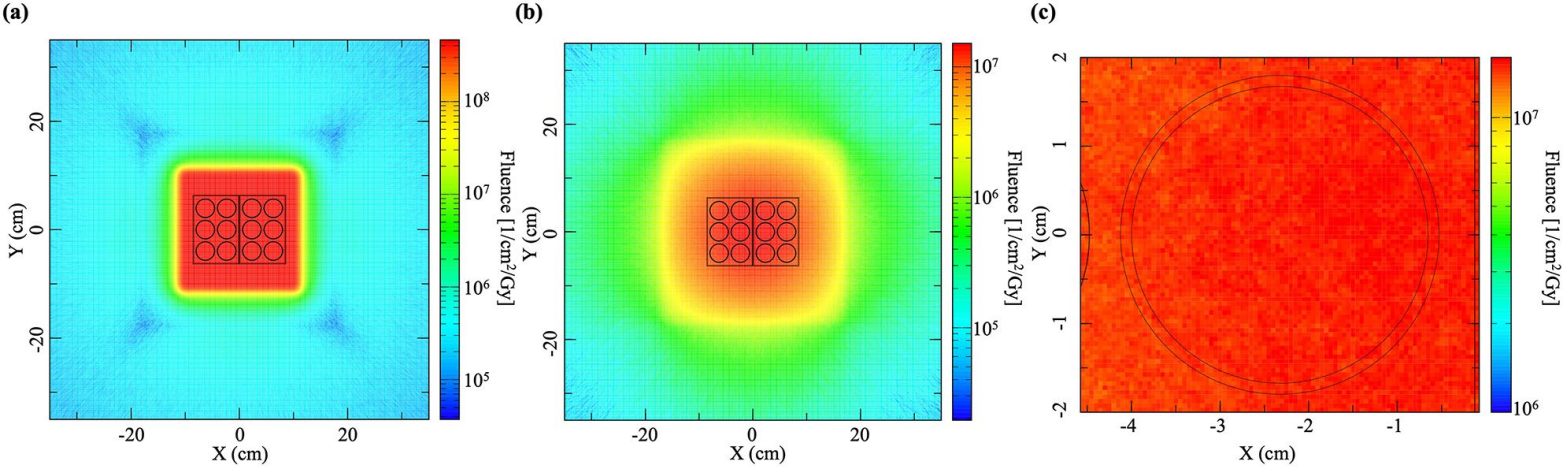
Dose distributions of the spread-out of Bragg peak central cross section calculated using Monte Carlo simulations. (**a**) The proton beam dose distributions of irradiation field. (**b**) The neutron dose distributions of irradiation field. (**c**) The neutron dose distributions in a well of 6-well tissue culture plates.

The dose distributions of proton and neutron in the irradiation field and the dose distributions of neutrons in a well of the 6-well tissue culture plates calculated using Monte Carlo simulations are shown in Fig. 1. The mean neutron fluence in a well of 6-well tissue culture plates was 1.38 × 10^7^ (range: 1.28 × 10^7^–1.51 × 10^7^) (1/cm^2^/Gy) and the neutron distribution in the well was found to be within ± 10% of the mean neutron fluence. Proton beams and neutrons were confirmed to be uniformly irradiated on the 6-well tissue culture plates (Fig. 1).

### Intracellular boron concentration

The intracellular boron concentration of each cell is shown in Table 1. The ratios of intra- and extracellular boron concentrations were 3.69–4.55 in 20 ppm of boron and 2.11–4.24 in 80 ppm of boron.

**Table 1:** Intracellular boron concentration and the ratio of intra- and extracellular boron concentrations.

Extracellular boron concentration	HSG	MG63	SAS	G-361
20 ppm, mean (range)	73.7 (73.3–73.8)	87.8 (85.2–91.7)	90.9 (80.2–103.7)	85.7 (80.6–91.4)
I/E ratio	3.69	4.39	4.55	4.28
80 ppm, mean (range)	168.7 (166.3–173.0)	230.1 (228.8–231.2)	193.6 (131.9–227.6)	339.0 (295.4–386.9)
I/E ratio	2.11	2.88	2.42	4.24

*G-361* human malignant melanoma cell, *HSG* human salivary gland tumor cell, *I/E* intra- and extracellular boron concentration, *MG63* human osteosarcoma cell, *SAS* human tongue squamous-cell carcinoma cell.

### Cell-survival curve

**Fig. 2:**
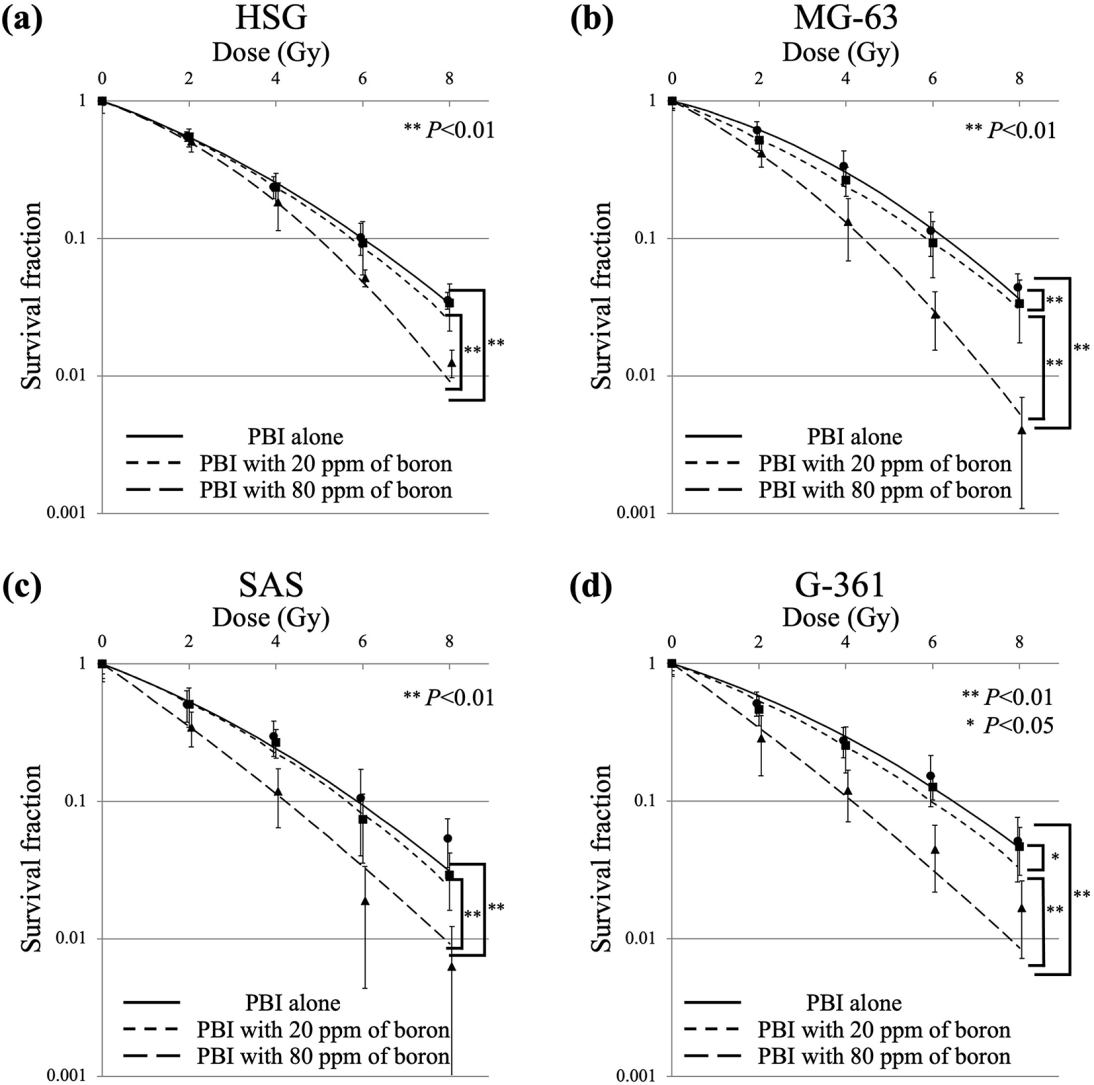
Survival curves for (**a**) HSG, (**b**) MG63, (**c**) SAS, and (**d**) G-361 cells after proton beam irradiation alone (solid line), proton beam irradiation with 20 ppm of boron (dash-dotted line), and proton beam irradiation with 80 ppm of boron (dashed line). Data are presented as the mean ± standard deviation, fitted to the linear-quadratic model. Significantly different at * *P* < 0.05 and ** *P* < 0.01. *G-361* human malignant melanoma cell, *HSG* human salivary gland tumor cell, *MG63* human osteosarcoma cell, *PBI* proton beam irradiation, *SAS* human tongue squamous-cell carcinoma cell.

**Fig. 3:**
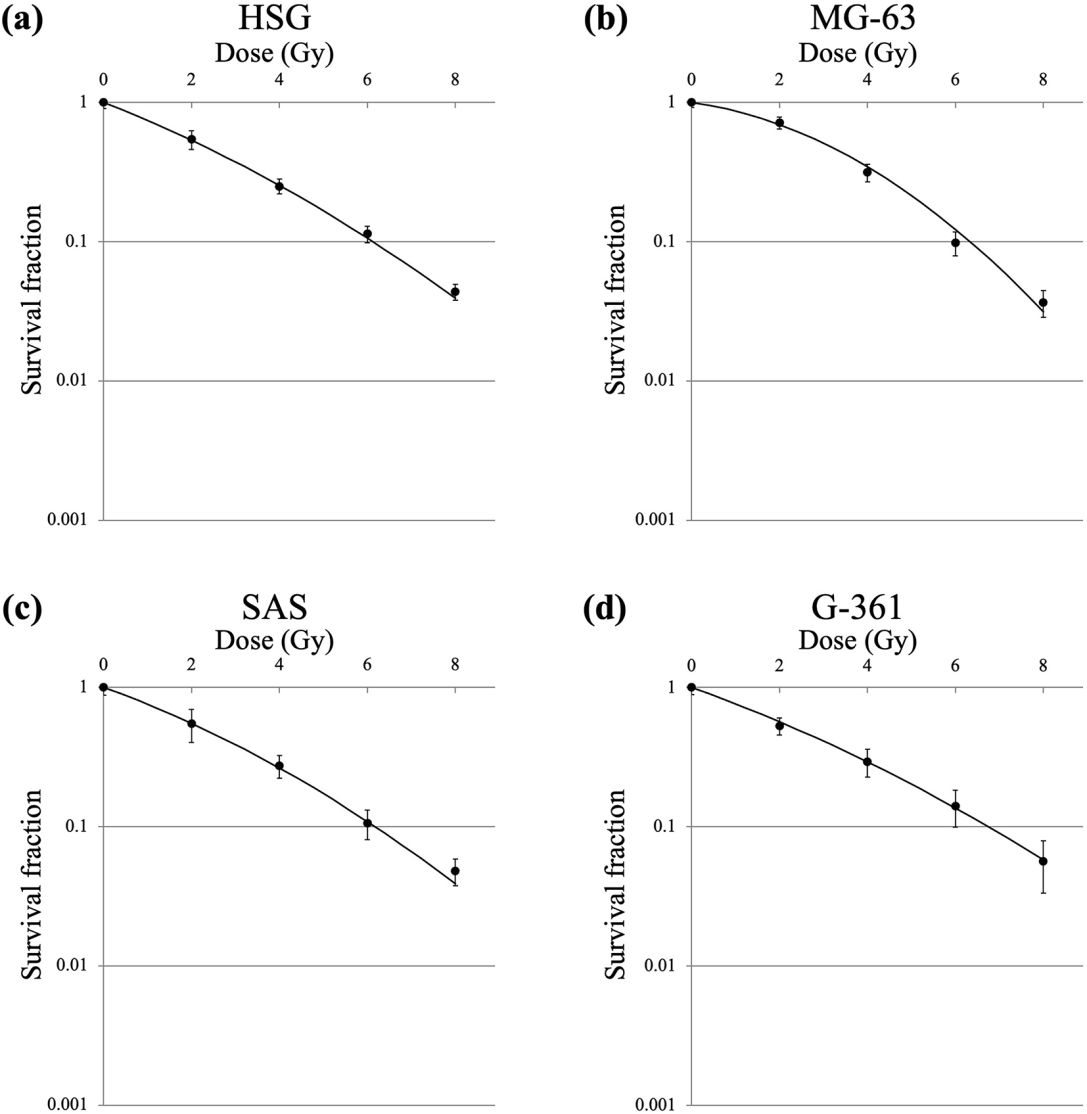
Survival curves for (**a**) HSG, (**b**) MG63, (**c**) SAS, and (**d**) G-361 cells after X-ray irradiation. Data are presented as the mean ± standard deviation, fitted to the linear-quadratic model. *G-361* human malignant melanoma cell, *HSG* human salivary gland tumor cell, *MG63* human osteosarcoma cell, *SAS* human tongue squamous-cell carcinoma cell.

The survival curves under different irradiation schemes of proton beam irradiation and X-ray irradiation in HSG, MG63, SAS, and G-361 cells are shown in Figs. 2 and 3, respectively. The horizontal axis in Fig. 2 presents the physical dose calculated using the treatment planning system of PBT and does not include the additional dose generated using BNCR. D_10_ of proton beams alone, proton beams with 20 ppm of boron, proton beams with 80 ppm of boron, and X-rays in the four cell lines are shown in Table 2. The RBE values of proton beams alone, proton beams with 20 ppm of boron, and proton beams with 80 ppm of boron for the four cell lines are shown in Table 2 and SER values of proton beams with 20 ppm of boron and proton beams with 80 ppm of boron for the four cell lines are shown in Table 2.

**Table 2:** D_10_, RBE, and SER in each cell.

	D_10_ (Gy)	RBE	SER
HSG			
X-rays	6.12		
Proton beams alone	6.00	1.02	
Proton beams with 20 ppm of boron	5.74	1.07	1.04
Proton beams with 80 ppm of boron	4.98	1.23	1.21
MG63			
X-rays	6.33		
Proton beams alone	6.29	1.01	
Proton beams with 20 ppm of boron	5.88	1.08	1.07
Proton beams with 80 ppm of boron	4.39	1.44	1.43
SAS			
X-rays	6.17		
Proton beams alone	5.87	1.05	
Proton beams with 20 ppm of boron	5.64	1.09	1.04
Proton beams with 80 ppm of boron	4.21	1.46	1.39
G-361			
X-rays	6.74		
Proton beams alone	6.47	1.04	
Proton beams with 20 ppm of boron	5.99	1.13	1.08
Proton beams with 80 ppm of boron	4.13	1.63	1.57

*D*_*10*_ dose that resulted in a surviving fraction of 10%, *G-361* human malignant melanoma cell, *HSG* human salivary gland tumor cell, *MG63* human osteosarcoma cell, *RBE* relative biological effectiveness, *SAS* human tongue squamous-cell carcinoma cell, *SER* sensitizer enhancement ratio.

Comparison of the cell survival fraction for HSG cells under different irradiation schemes of proton beam and X-ray irradiation showed that proton beams with 80 ppm of boron significantly reduced the cell survival compared with X-ray irradiation, proton beams alone, and proton beams with 20 ppm of boron (*P* < 0.01, *P* < 0.01, and *P* < 0.01, respectively); however, no significant differences were noted between X-ray irradiation and proton beams alone (*P* = 0.22), X-ray irradiation and proton beams with 20 ppm of boron (*P* = 0.15), and proton beams alone and proton beams with 20 ppm of boron (*P* = 0.60). In MG63 cells, proton beams with 80 ppm of boron significantly reduced the cell survival fraction compared with X-ray irradiation, proton beams alone, and proton beams with 20 ppm of boron (*P* < 0.01, *P* < 0.01, and *P* < 0.01, respectively), and proton beams with 20 ppm of boron significantly reduced the cell survival fraction compared with X-ray irradiation and proton beams alone (*P* < 0.01 and *P* < 0.01, respectively); however, no significant difference was noted between X-ray irradiation and proton beams alone (*P* = 0.28). In SAS cells, proton beams with 80 ppm of boron significantly reduced the cell survival fraction compared with X-ray irradiation, proton beams alone, and proton beams with 20 ppm of boron (*P* < 0.01, *P* < 0.01, and *P* < 0.01, respectively); however, no significant differences were noted between X-ray irradiation and proton beams alone (*P* = 0.46), X-ray irradiation and proton beams with 20 ppm of boron (*P* = 0.11), and proton beams alone and proton beams with 20 ppm of boron (*P* = 0.45). In G-361 cells, proton beams with 80 ppm of boron significantly reduced the cell survival fraction compared with X-ray irradiation, proton beams alone, and proton beams with 20 ppm of boron (*P* < 0.001, *P* < 0.01, and *P* < 0.01, respectively), and proton beams with 20 ppm of boron significantly reduced the cell survival fractions compared with X-ray irradiation (*P* < 0.05); however, no significant difference was noted between X-ray irradiation and proton beams alone (*P* = 0.57), proton beams alone and proton beams with 20 ppm of boron (*P* = 0.15).

MG63 and G-361 cell lines showed higher SER than HSG and SAS cell lines. Additionally, a significant positive correlation was noted between intracellular boron concentration and SER values (*R* = 0.97, *P* < 0.01).

## Discussion

We confirmed the generation of neutrons by proton beam irradiation using Monte Carlo simulations and showed an increase in the cell killing effect of NCEPBT using in vitro experiments. The cell killing effect was particularly enhanced in sarcomas and melanomas, which are considered radioresistant tumors. Additionally, intracellular boron increased the cell killing effect in a concentration-dependent manner. BPA can theoretically increase the cell killing effect in tumors because of its tumor-selective uptake by LAT1. Therefore, we believe that these results will improve the weak points of PBT, that is, the difficulty in increasing the administration dose for tumors and low efficacy against radioresistant tumors. Furthermore, NCEPBT could have used the thermal neutron component generated by proton beam irradiation to increase the tumor dose.

In the present study, RBE and intracellular boron concentration might be key factors. High LET radiation is highly effective in radioresistant tumors^16–18^. In the high LET radiation modality of C-ion RT, a previous study showed that osteosarcoma cells (U2OS, one of the radioresistant tumor cell lines) have a higher RBE than HSG (normal radiosensitivity tumor cell line)^19^. The LET of alpha particles and lithium ions generated using BNCR are 163 and 210 keV/µm, respectively, which are high LET radiations and may have resulted in high RBE and increased biological dose in radioresistant tumor cells compared to normal radiosensitivity tumor cells. Therefore, in the present study on NCEPBT, the sensitizing effect may also be higher for radioresistant tumors than for normal radiosensitivity tumor cells. However, although RBE may be important as a factor affecting the sensitizing effect, the RBE of BNCR in various cell lines is not yet known and the RBE of BNCR should be investigated in the future.

Concerning intracellular boron concentrations, all cell lines had 2–4.5-fold the extracellular boron concentration. While previous reports showed a linear relationship between intracellular and extracellular boron concentrations^20^, the present study showed that higher boron concentrations resulted in a lower intra- and extracellular boron concentration ratio except for that in G-361 cells. In contrast, when the extracellular boron concentration was 80 ppm in HSG, MG63, and SAS, it was 2–3-fold, similar to that reported previously^20^. In the present study, SER values showed a strong correlation with intracellular boron concentration, with higher intracellular boron concentrations indicating higher SER. Previous studies have demonstrated a relationship between LAT1 and BPA uptake^8^ and highlighted the fact that the strength of LAT1 expression strongly affects the BPA uptake ability of tumor cells^21^. However, LAT1 expression in the cell lines used in the present study was positive for G-361, but is otherwise unknown^22^. It would be advisable to confirm the up-regulation of LAT1 before NCEPBT in the future.

BPA has already been used in the clinical practice of BNCT, and its administration to the human body may be feasible. Although we would like to apply BPA to PBT for NCEPBT in the future, there are several issues to be solved. The first is that the dose fractionation in PBT is 10–25 fractions, which is larger than that in BNCT, and administering the drug after each irradiation reflects physical and cost burden. Second, the blood concentration of boron administered to humans during BNCT is approximately 30 ppm by the 3-hour infusion of 500 mg of BPA/kg body weight^23^, and the sensitization effect is not satisfactory when considered as a concentration close to the extracellular boron concentration of 20 ppm in the present study. With regard to the high doses of BPA administered, a previous report showed a 6-hour infusion of 900 mg of BPA/kg body weight in BNCT for glioblastoma multiforme was not associated with any severe toxicity associated with the administration of BPA^24^. However, NCEPBT may require daily administration, which is physically burdensome and would be difficult to administer in the manner currently possible with BNCT. In the future, it is necessary to develop boron agents that can stay at high concentrations longer in the cell during NCEPBT.

We prepared the dose distributions of proton and neutron beams calculated using Monte Carlo simulations for the confirmation of proton and neutron distribution and the prediction of an increase in the cell killing effect of NCEPBT. However, the fluence of neutrons was not consistent with the previously reported data^12^ and the accurate calculation of the alpha particles and lithium ions doses generated using BNCR was difficult and could not be calculated in the present study. As few reports exist on the fluence of neutrons generated after proton beam irradiation calculated using Monte Carlo simulations and actual neutron fluence measurement is difficult, determining the correct report, either the previous or current one, is impossible. Furthermore, an increase in the cell killing effect of NCEPBT is also related to the RBE of the alpha particles and lithium ions. Therefore, currently predicting an increase in the cell killing effect of NCEPBT from Monte Carlo simulations is difficult. Accurate calculation using Monte Carlo simulations in the fluence of neutrons, and the dose and RBE of alpha particles and lithium ions generated after BNCR are further issues to be resolved.

In other particle therapy, C-ion beams are used in clinical practice. C-ion beam irradiation generates neutrons owing to nuclear reactions such as proton beam irradiation. Neutron capture enhanced C-ion RT has been reported previously, and the results are similar to those of the current NCEPBT^25^. In contrast, if the biological dose is matched with proton beam irradiation, the physical dose of C-ion beams is lower than that of proton beams because C-ion beams have higher RBE. Therefore, for the same biological dose, C-ion beams generate fewer neutrons than proton beams^12^ and to achieve the same sensitizing effect, the neutron capture enhanced C-ion RT requires a higher boron concentration than the NCEPBT. Also, C-ion RT shows a favorable cell killing effect in radioresistant tumors because of their higher LET^16,19^. Therefore, for these reasons, we considered that neutron capture enhanced C-ion RT would increase the dose in the tumor than C-ion RT alone, but the neutron capture enhanced C-ion RT would not benefit as much as the NCEPBT.

Our study had certain limitations. First, we performed our study on only four cell lines and not on cell lines from diseases that are amenable to PBT, such as hepatocellular carcinoma and brain tumors. However, we believe that the present study is sufficient as the first confirmation of the increase in the cell killing effect of NCEPBT. Second, high concentrations of boron administered to cells may be difficult to administer to the human body. We look forward to future chemical development as we eventually aim for its clinical use. Third, the present study was conducted only in vitro. For clinical application, it may be necessary to conduct the study in vivo to confirm the tumor and healthy tissue response and to confirm the distribution of the boron agents at high concentrations. Additionally, whether neutron distribution in vivo can be calculated is not clear yet; thus, an in vivo study of NCEPBT is a potential goal for future research. Furthermore, we only calculated the neutron fluence, and accurate dose calculation of NCEPBT was not available in the present study. Because slight amounts of BPA are taken up by healthy tissues, BNCR occurs in these tissues in NCEPBT. For clinical use, accurate dosimetry of tumors and healthy tissues is essential for predicting treatment efficacy and adverse events. Therefore, accurate dose calculation for NCEPBT is one of the key requirements for its clinical application and the next issue that we are focusing on.

## Conclusion

We showed an increase in the cell killing effect of NCEPBT, and higher boron concentration resulted in higher sensitization. Our results encourage further investigation of the NCEPBT, such as using other cell lines and in vivo experiments, and should aid the clinical use of NCEPBT.

## Data Availability

The datasets generated for this study are available to the corresponding author upon request.
